# Two new species of forget-me-nots ( *Myosotis*, Boraginaceae) from New Zealand

**DOI:** 10.3897/phytokeys.16.3602

**Published:** 2012-08-21

**Authors:** Carlos A. Lehnebach

**Affiliations:** 1 Museum of New Zealand Te Papa Tongarewa, POBOX 467, Wellington, New Zealand

**Keywords:** Boraginaceae, Kahurangi National Park, *Myosotis*, *Myosotis chaffeyorum*, *Myosotis mooreana*, new species, New Zealand, New Zealand Flora

## Abstract

Two new species of forget-me-nots, *Myosotis chaffeyorum* C.A.Lehnebach **sp. nov.** and *Myosotis mooreana* C.A.Lehnebach **sp. nov.** are described and illustrated. These species are endemic to New Zealand and restricted to the mountains of north-west Nelson (South Island). Both species are uncommon and *Myosotis mooreana* is known from the type locality only. Their conservation status is rated as “Nationally Critical”. A table listing differences between these two new species and other similar species and an identification key are provided.

## Introduction

The genus *Myosotis* L. is found in the Northern and Southern Hemisphere. Phylogenetic analyses of nuclear and chloroplast sequences suggest *Myosotis* originated in the Northern Hemisphere (Winkworth et al. 2002) and is nested within the tribe Cynoglosseae along with *Trigonotis* Steven (Weigend et al. 2010). In the Southern Hemisphere, New Zealand is the centre of diversity for the genus *Myosotis*, and 39 species have been listed for the country (Breitwieser et al. 2012). These species occupy a great diversity of habitats ranging from coastal cliff faces and sand dunes to stream banks in forests, tussock grasslands, tarns, limestone and marble outcrops, and scree-slopes on the mountains of the Southern Alps. This ecological diversification is matched by an equally remarkable diversification of habit (cushion, single rosette, creeping/trailing stems), plant size (less than a centimetre to up to 40 cm height), and vegetative (leaf shape, indumentum type) and reproductive characters (white, yellow, brown-bronze, blue flowers and automatic selfing and out-crossing species).

Despite the many phenotypic differences exhibited by New Zealand *Myosotis*, identification at the species level can be challenging, especially when dealing with rare or under-collected species or poorly press-dried specimens in which flower and leaf characters have become distorted. The former situation, in particular, is commonly encountered by students of this genus, as many species have a highly restricted distribution with small-size populations (Brandon 2001). In fact, some of the currently recognised species are known only from a single locality (e.g. *Myosotis alboserica* Hook.f., *Myosotis laeta* Cheeseman) or, in more extreme cases, the type collection is the only collection ever made (e.g. *Myosotis laingii* Cheeseman).

Currently, a revision of the genus *Myosotis* in New Zealand is underway and species limits for a number of widespread and critically endangered species are under assessment. Also, phylogenetic affinities within New Zealand species are being studied using DNA sequences of both nuclear and chloroplast markers and AFLP fingerprinting data (Meudt et al. in preparation). Towards this end, a number of collecting trips to sub-alpine and alpine areas of the North and South Island of New Zealand have taken place between 2009 and 2012. An expedition to Kahurangi National Park in north-west Nelson (South Island, 41°11'26"S, 172°44'52"E), one of the diversity hotspots for *Myosotis* in the country, uncovered the existence of two morphologically and ecologically distinct entities, which are illustrated and described here as new species. An identification key and a table summarising morphological differences between these two new species and other morphologically similar species are included.

## Methods

Specimens were press-dried in the field as soon as they were collected. Flowers, when available, were collected and preserved in 70% ethanol for later examination. Some samples were kept fresh and later photographed under a dissecting microscope. To test whether phenotypic differences observed in the field were due to phenotypic plasticity, some samples were cultivated for two seasons, under common garden conditions. Vegetative and reproductive characters were observed and measured from fresh and press-dried material. These records were later compared with descriptions in Moore (1961, 1988), Moore and Simpson (1973), and with fresh and dried material of morphologically similar species, i.e. *Myosotis brockiei* L.B.Moore & M.J.A.Simpson, *Myosotis forsteri* Lehm., *Myosotis matthewsii* L.B.Moore, and *Myosotis spathulata* G.Forst. (Table 1). Collections of *Myosotis* from the Allan Herbarium (CHR), the Department of Conservation Nelson-Marlborough Conservancy (NM) and the Museum of New Zealand Te Papa Tongarewa (WELT) were checked for previous collections of these two entities. Type material of known *Myosotis* species stored at the Auckland Museum (http://www.aucklandmuseum.com/57/botany ) and Museum of New Zealand (http://collections.tepapa.govt .nz/search.aspx?term=Myosotis types) were also studied and compared with the new material collected at Kahurangi National Park.

**Table 1.** Morphological comparison of *Myosotis chaffeyorum*, *Myosotis mooreana* with other species of *Myosotis*. Data for *Myosotis brockiei*, *Myosotis forsteri*, *Myosotis matthewsii*, and *Myosotis spathulata* are from Moore (1961, 1988), Moore and Simpson (1973) and representative voucher specimens (Appendix 1).

Character	*Myosotis chaffeyorum*	*Myosotis matthewsii*	*Myosotis spathulata*	*Myosotis mooreana*	*Myosotis brockiei*	*Myosotis forsteri*
Rosette leaf lamina shape	orbicular	orbicular	orbicular to broadly elliptic	obovate	narrow-elliptic	orbicular to broadly elliptic
Rosette leaf lamina size (mm)	8.1 – 8.9 × 7.7 – 9.2	16.8 – 21 × 15.3 – 20.3	10.1 – 16.3 × 9 – 12.2	32.4 – 56 × 15.8 – 20.5	60 – 120 × 10 – 15	25 – 30 × 31 – 39
Leaf indumentum	strigose	hispid	hispid	hispid	tomentose	hispid
Hairs on leaf margin	appressed	appressed	arcuate	arcuate/straight	arcuate/erect	arcuate
Rosette leaf petiole width (mm)	0.2 – 0.3	0.5 – 0.6	0.3 – 0.4	1.4 – 1.8	1.8 – 1.9	1.9 – 3
Hairs on petiole	appressed	appressed	erect/arcuate	arcuate	arcuate	erect/arcuate
Stem habit	decumbent	decumbent	creeping	ascending	erect	ascending
Hairs on stem	appressed only	appressed only	erect/arcuate	appressed, arcuate, erect	appressed, arcuate, erect	arcuate/appressed
Stem rooting at nodes	no	no	yes	no	no	no
Flower arrangement	solitary, usually opposite to leaf	ill-defined cyme, on stem below/above leaf	solitary, on stem above/below leaf	cyme	cyme	cyme
Pedicel at fruiting (mm)	1.5 – 3	4.6 – 8.4	2.3 – 3.7	2.5 – 2.9	3.6 – 4.3	2.6 – 3
Corolla diameter (mm)	3.7 – 4	5 – 8	3	3.8 – 4.8	9 – 10	3.2 – 4.6
Position of anthers	within corolla tube	outside corolla tube	within corolla tube	within corolla tube	outside corolla tube	within corolla tube
Fruiting calyx (mm)	2.3 – 3	3.5 – 4.2	2.1 – 2.7	2.7 – 2.8	3.9 – 4.4	4.5 – 5
Nutlet shape	ovoid-ellipsoid	ovoid-ellipsoid	ovoid	ovoid-ellipsoid	ovoid	ovoid
Nutlet size (mm)	1.2 × 0.7 – 0.8	1.2 – 1.8 × 0.8 – 1.2	1 – 1.3 × 0.6 – 1	1.4 – 1.5 × 0.7 –0.9	1.7 – 1.9 × 1.1	1.2 – 1.8 × 0.9 – 1.1
Known distribution	South Island	North Island	North & South Island	South Island	South Island	North & South Island
Representative specimen	CHR 311719	WELT SP093687	WELT SP090633	WELT SP092756	WELT SP090249	WELT SP092226

## Taxonomy

### Myosotis chaffeyorum

C.A.Lehnebach sp. nov.

urn:lsid:ipni.org:names:77121627-1

http://species-id.net/wiki/Myosotis_chaffeyorum

Figs 1 –2

#### Diagnosis.

Similar to *Myosotis spathulata* and *Myosotis matthewsii***,** but differs from *Myosotis spathulata* by its well-defined, slender petiole; appressed, straight hairs on stem, petiole, leaf lamina and margin; and its decumbent stem not rooting at nodes. It differs from *Myosotis matthewsii* by its strigose indumentum on leaf upper surface; smaller flowers (3.7 – 4 mm across vs 5 – 8 mm across); stamens included in the corolla tube and fruiting calyx on shorter pedicel (1.5 – 3 mm vs 4.6 – 8.4 mm).

#### Type.

**Figure 1.**
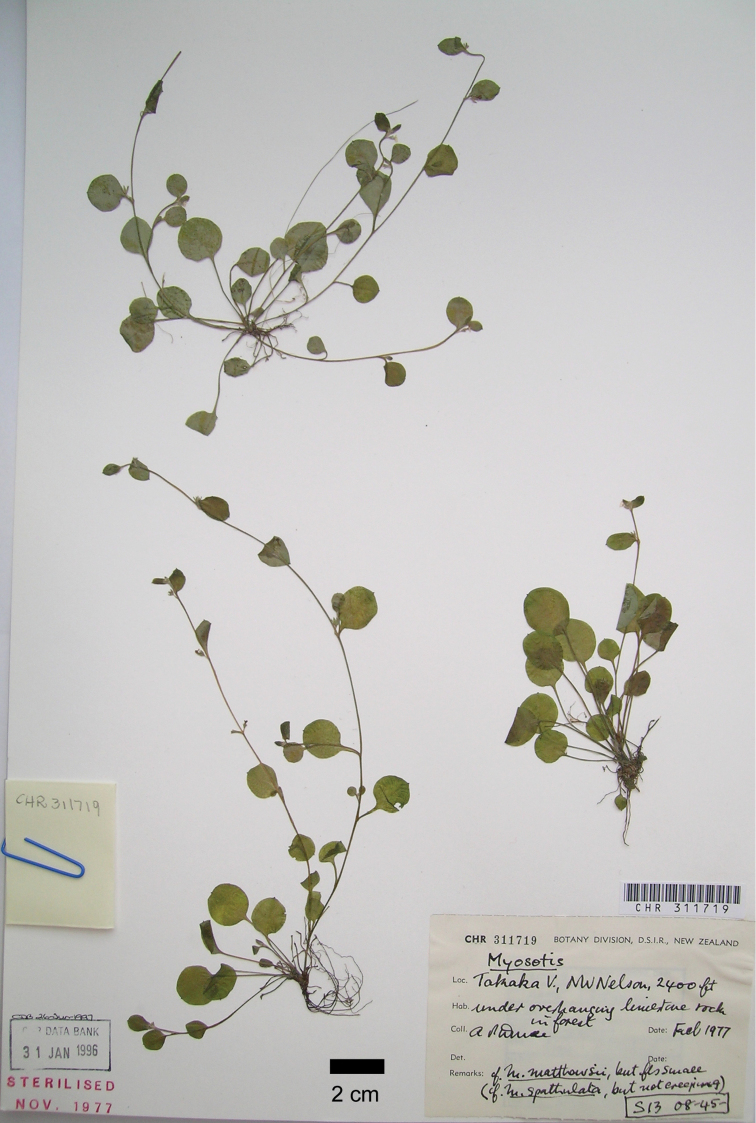
Photograph of the holotype of *Myosotis chaffeyorum* C.A. Lehnebach. (A.P.Druce s.n., CHR 311719). Copyright Allan Herbarium (CHR), Landcare Research. New Zealand.

New Zealand. South Island, north-west Nelson: Takaka Valley, under overhanging limestone rock in forest, alt. ca 730 m, February 1977, A.P.Druce s.n. (Holotype: CHR [CHR 311719]; Fig. 1).

Plant perennial, 1.4 – 4.6 cm tall. Rosette leaves 6 – 9, lamina orbicular, 8.1 – 8.9 × 7.7 – 9.2 mm, apex mucronate, mucro ca 1 mm long. Leaf indumentum strigose, hairs on upper and lower surface sparsely distributed, non overlapping, appressed and antrorse. Hairs on leaf margin appressed. Petiole well-defined, 11.9 – 13.5 × 0.2 – 0.3 mm; hairs appressed, antrorse, sparsely distributed. Stem light brown, decumbent, not rooting at nodes, 59 – 100 × 0.3 – 0.5 mm. Stem hairs appressed as for leaves, hairs 0.2 – 0.6 mm long. Stem leaves elliptic or orbicular, 7.2 – 8.8 × 4.4 – 6.2 mm, mucronulate, shortly petiolate or sessile towards distal end of stem. Indumentum as for rosette leaves. Calyx lobes lanceolate, 1.5 – 2 mm long, hairs in upper half of the lobe only. Fruiting calyx 2.3 – 3 mm long; hairs appressed, straight, overlapping, not uniform in size. Flowers borne along the trailing stem, each usually opposite to a leaf. Pedicel at fruiting 1.5 – 3 mm long. Corolla white with yellow scales, 3.7 – 4 mm across. Corolla lobes obovate, not overlapping, 1.4 – 1.8 × 1.2 – 1.6 mm, apex rounded or irregularly notched. Corolla tube 2 – 2.2 mm long. Stamens included in the corolla, with only the anther’s appendage slightly above the scales. Filament attached below scales. Anther 0.5 × 0.2 mm. Style 1.8 mm long, stigma clavate. Nutlet smooth, light brown, ovoid to ellipsoid, 1.2 × 0.7 – 0.8 mm, ventral surface rounded.

#### Specimens examined.

New Zealand, South Island: north-west Nelson. Kahurangi National Park, upper Takaka River track, under overhang of limestone outcrop, alt. 857 m, 8 January 2011, C.A.Lehnebach & A.Zeller s.n. (WELT SP092173). Track along Takaka River, under overhang of limestone, alt. 736 m, 8 January 2011, C.A.Lehnebach & A.Zeller s.n. (WELT SP092172). Takaka Valley, alt. 900 m, under overhanging limestone rock in forest, February 1977, A.P. Druce s.n. (CHR 311720). Takaka River, near Ghost Creek Saddle, alt. 840 m, under overhang of limestone outcrop on dry loose fine soil, 22 April 2005, S. Courtney s.n. (NM 2688). Takaka Valley, Paynes Ford, at a base of limestone bluff, 30 October 2010, S. Courtney s.n. (NM 4835). Aniseed Valley. Valley down which Roding River, tributary of the Wairoa River, runs, 26 November 1967, R.H.S. (CHR 269160).

**Figure 2.**
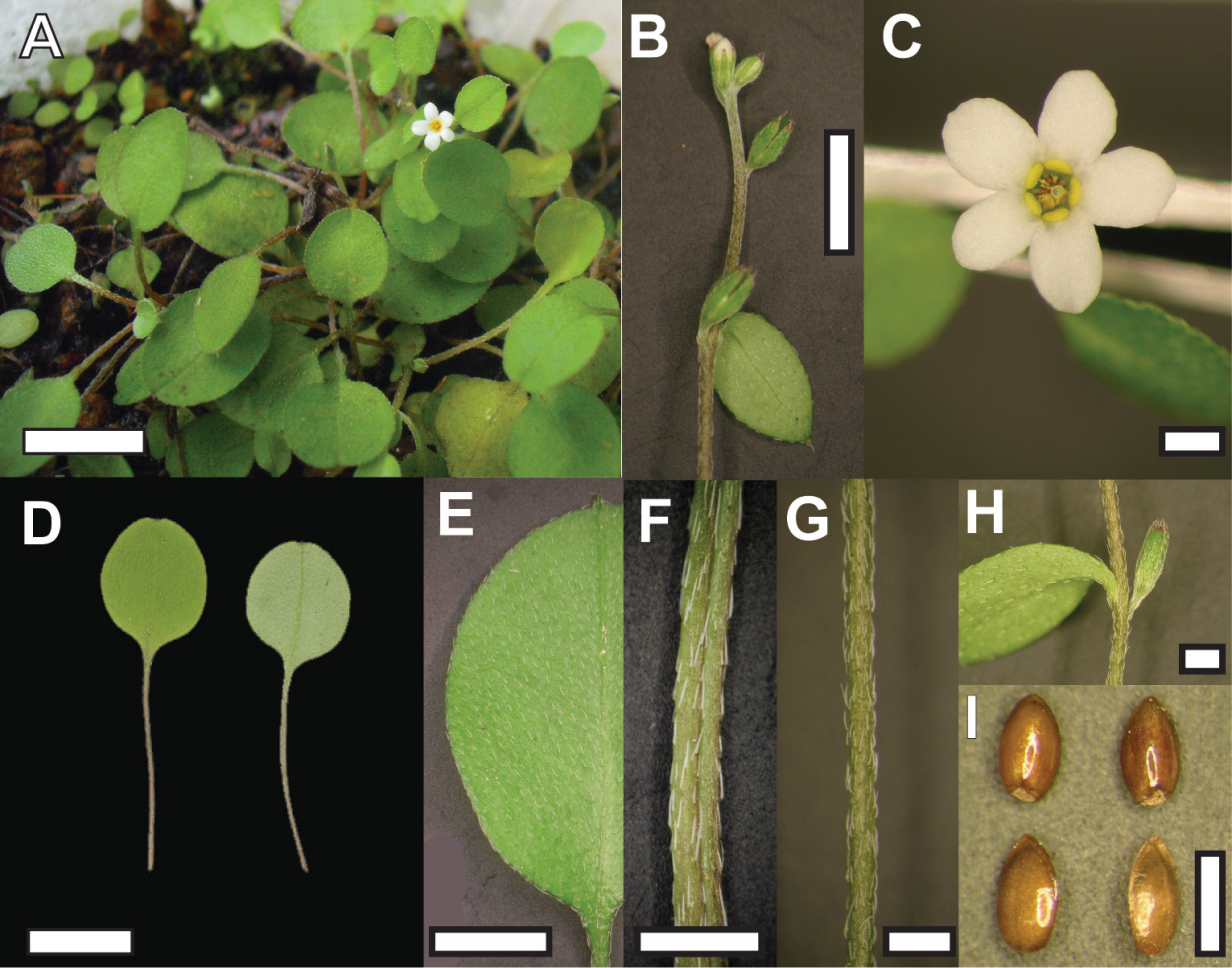
Plant of *Myosotis chaffeyorum* (**A**) and close-up view of vegetative and reproductive structures (**B** Flowering stem **C** Flower **D** Rosette leaves **E** Rosette leaf indumentum **F** Petiole **G** Stem **H** Node with flower and stem leaf **I** Nutlets). Bar = 1 cm in A & D, 5 mm in B & E, 1 mm in C, F, G, H, I. Material from WELT SP092173 (D, E, F, G, H ) and WELT SP094151 (A, B, C, I).

#### Etymology.

This species is named after Annie and Henry Chaffey (http://www.teara.govt.nz/en/biographies/4c15/1 ), who lived from ca 1913, for almost 40 years, as self-sustained, pioneers/exiles in this mountainous area of Kahurangi National Park. The tiny cottage they lived in still remains in place and served me as shelter during the expedition in which this new species was collected.

#### Distribution.

Endemic to New Zealand, only found in north-west Nelson (South Island). Detailed geographic coordinates are not shown to protect this species from illegal collection but are available from the author on request.

#### Conservation status.

Currently the existence of only five populations of this species can be confirmed. Two of them are within Kahurangi National Park where they occupy an approximate area of 6 m^2^ and 1 m^2^. The number of individuals at each site is low, ca 20 and 3, respectively. Three other populations are found outside the park, two of them in smaller protected areas which are regularly monitored by the Department of Conservation. At all these sites the species occupies a similar habitat and a small area (Shannel Courtney, personal communication). Considering this information and following the New Zealand Threat Classification System (Townsend et al. 2008), *Myosotis chaffeyorum* should be considered as “Nationally Critical”.

#### Ecology.

This is a perennial, self-pollinating species. It is habitat-specific and restricted to dry loose fine soil under shelters formed by limestone overhangs.

#### Discussion.

The earliest known collection of *Myosotis chaffeyorum* dates from 1967 (CHR 269160). Its distinctiveness from other species of *Myosotis*, however, was only noticed ca 10 years later when Anthony (Tony) Druce collected it while surveying the flora associated with Palaeogene calcareous rocks in north-west Nelson (Druce, unpublished). Druce noticed the overall similarity of this species to *Myosotis matthewsii* and *Myosotis spathulata* but also differences between them, which he wrote on the labels of the herbarium sheets of these collections (see Fig. 1). These notes read “cf. *Myosotis matthewsii*, but the flowers are smaller” and “cf. *Myosotis spathulata*, but stem not creeping”.

*Myosotis matthewsii* and *Myosotis spathulata* are the only forget-me-nots in New Zealand with orbicular rosette leaves with which *Myosotis chaffeyorum* could be confused. The small flower with stamens within the corolla tube, the absence of roots at the nodes of the decumbent flowering stem and the strigose indumentum of the leaf lamina, petiole and stems of *Myosotis chaffeyorum* are key diagnostic characters to distinguish it from *Myosotis matthewsii* or *Myosotis spathulata*. In the absence of flowers, *Myosotis matthewsii* can be distinguished from *Myosotis chaffeyorum* by the presence of arcuate and erect hairs on the upper surface of the rosette leaves, larger leaves and a longer pedicel at fruiting (see Table 1). As for *Myosotis spathulata*, if no stems are present, it can be readily distinguished from *Myosotis chaffeyorum* by the hispid indumentum on the leaf petiole and larger leaf lamina (see Table 1).

### Myosotis mooreana

C.A.Lehnebach sp. nov.

urn:lsid:ipni.org:names:77121628-1

http://species-id.net/wiki/Myosotis_mooreana

Figs 3 –4

#### Diagnosis.

Similar to *Myosotis forsteri* and *Myosotis brockiei*, but differs from *Myosotis forsteri* by its obovate rosette leaves; larger leaves at base of the cyme and smaller calyx at fruiting. It differs from *Myosotis brockiei* by its smaller flowers (3.8 – 4.8 mm vs 9 – 10 mm); stamens included within the corolla tube; fruiting calyx with shorter pedicel (2.5 – 2.9 mm vs 3.6 – 4.3 mm); and hispid indumentum on leaves and petiole.

#### Type.

**Figure 3.**
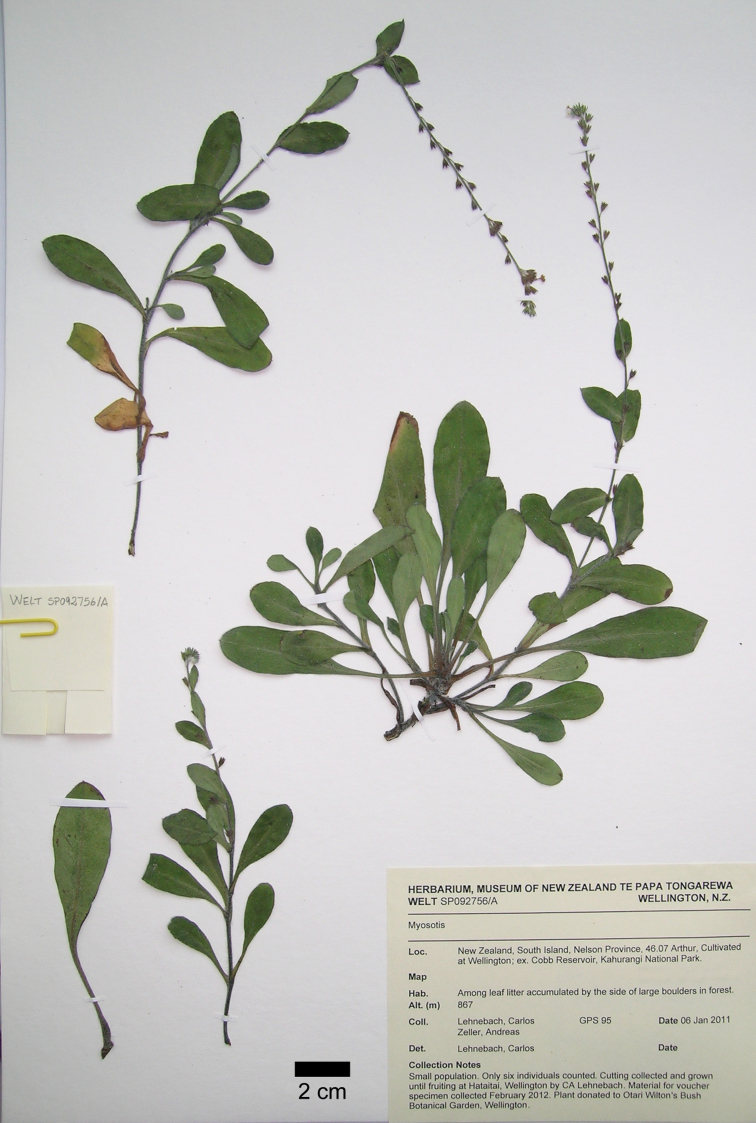
Photograph of the holotype of *Myosotis mooreana* C.A. Lehnebach. (C.A.Lehnebach & A.Zeller s.n., WELT SP092756/A).

NEW ZEALAND.South Island, north-west Nelson: Kahurangi National Park, Cobb Reservoir, among leaf litter accumulated by the side of large boulders in forest, alt. ca 867 m, 6 January 2011, C.A.Lehnebach & A.Zeller s.n. (Holotype: WELT [WELT SP092756/A]; Fig. 3).

Plant perennial, ca 20 cm tall. Rosette leaves 5 – 12, obovate, 32.4 – 56 ×15.8 – 20.5 mm; apex mucronulate, ca 0.4 mm long. Leaf lamina base attenuate to petiole. Leaf indumentum hispid, hairs on upper surface sparsely distributed, antrorse, arcuate or erect. Hairs on the lower surface sparsely distributed, retrorse, arcuate or erect. Hairs arcuate or straight on margin. Petiole 26 – 40 × 1.4 – 1.8 mm, hispid, hairs erect, arcuate, antrorse or retrorse on margins. Flowering stem ascending, dark green to brown, 210 × 1.5 – 1.9 mm. Hairs, appressed, arcuate or erect, 0.8 – 2.1 mm long. Stem leaves elliptic-obovate, mucronulate, 15.3 – 35.4 × 6.4 – 14 mm, either shortly petiolate or sessile towards distal end of the inflorescence. Indumentum as for rosette leaves. Cyme with 15 – 31 flowers. Pedicel at fruiting, 2.5 – 2.9 mm. Calyx lobes lanceolate, 2.3 – 2.8 mm long, hairs inside calyx in upper half or along entire lobe. Fruiting calyx 2.7 – 2.8 mm long, hairs densely distributed, overlapping and hooked or straight. Corolla white with yellow scales, 3.8 – 4.8 mm across, lobes ovate, 2 × 1.6 – 1.8 mm; not overlapping, apex rounded or irregularly notched. Corolla tube 3 mm long. Stamens included within the corolla tube, with only the anther’s appendage above the scales. Filament attached below the scales. Anther 0.8 × 0.2 mm. Style 3 mm long, stigma clavate. Nutlet smooth, dark brown, ovoid to ellipsoid, 1.4 – 1.5 × 0.7 – 0.9 mm, ventral surface keeled.

**Figure 4.**
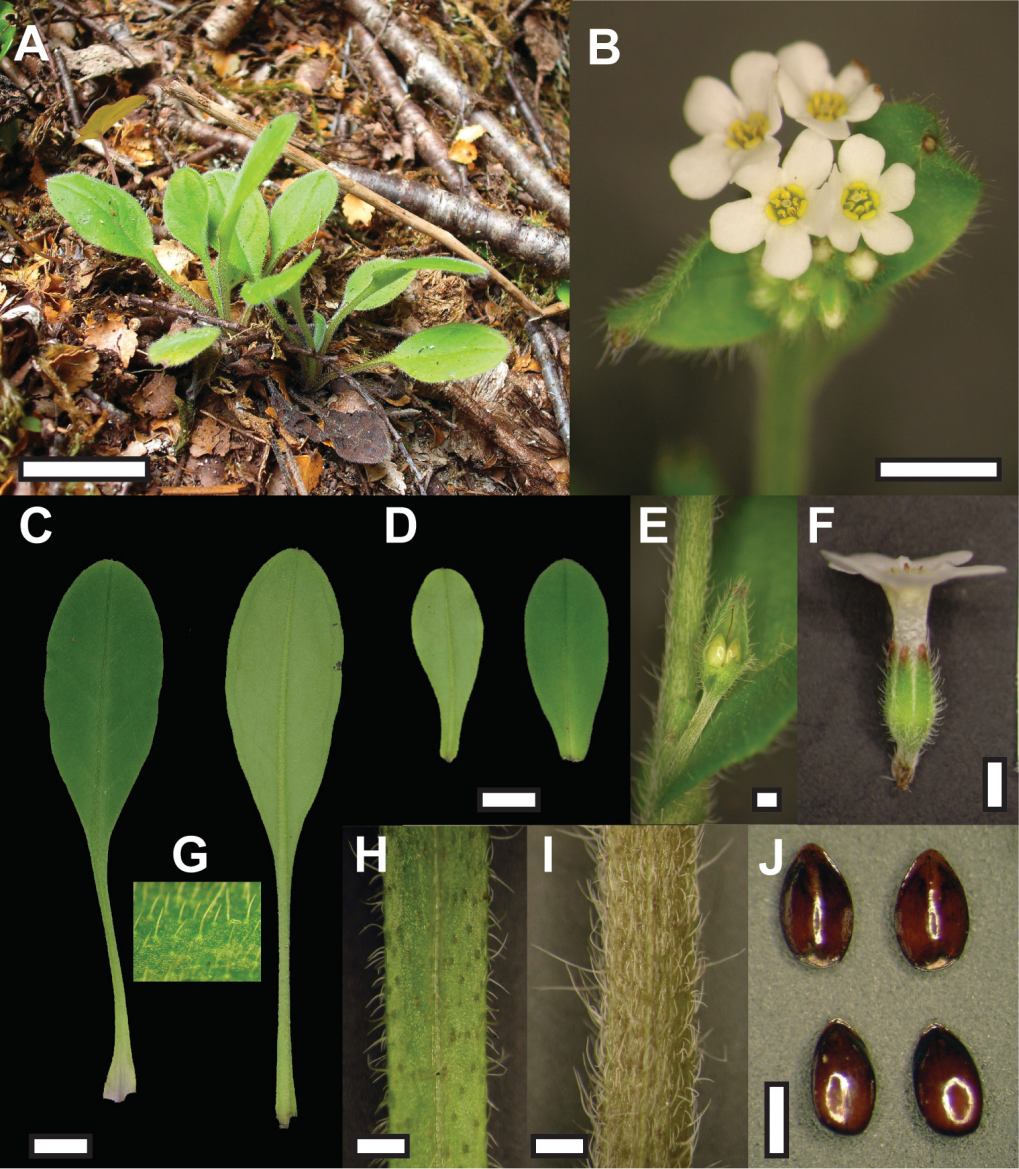
Plant of *Myosotis mooreana* in situ (**A**) and close-up view of vegetative and reproductive structures (**B** Inflorescence **C** Rosette leaves **D** Stem leaves **E** Fertilised flower at leaf axil **F** Flower **G** Rosette leaf indumentum, upper surface **H** Rosette leaf petiole **I** Stem **J** Nutlets). Bar = 1 cm in A, C & D, 5 mm in B, 1 mm in E, F, H, I, J. Material from WELT SP092756/A.

#### Etymology.

This species is named in honour of Lucy Beatrice Moore, New Zealand botanist (http://www.teara.govt.nz/en/biographies/5m55/1 ) who described several species of endemic *Myosotis* and revised this genus for the Flora of New Zealand (Allan 1961).

#### Distribution.

Endemic to New Zealand. Known from a single location in Kahurangi National Park, north-west Nelson (South Island). Detailed geographic coordinates are not shown to protect this species from illegal collection but are available from the author on request. It is likely *Myosotis mooreana* also occurs at two other sites within the Park (Shannel Courtney, personal communication) but this cannot be confirmed at this stage.

#### Conservation status.

Considering the small population size for this species (six individuals only), low number of mature individuals observed in the population and the small area of occupancy (less than 1m^2^), and following the New Zealand Threat Classification System (Townsend et al. 2008), *Myosotis mooreana* should be rated as “Nationally Critical”.

#### Ecology.

This is a perennial, fertile, self-pollinating species. It grows among the twigs and leaf-litter accumulated among large boulders under red beech (*Nothofagus fusca*) forest.

#### Discussion.

There are only two species of forget-me-nots in New Zealand that can be mistaken with *Myosotis mooreana*; i.e. *Myosotis brockiei* and *Myosotis forsteri*. These two are the only species growing in a similar habitat, under forest or scrub in north-west Nelson and throughout New Zealand, respectively. The main differences between *Myosotis mooreana* and *Myosotis brockiei* are the large flowers with stamens fully exserted beyond the corolla tube in the latter species. Flowers in *Myosotis mooreana* are only ca 5 mm across and stamens are always included within the corolla tube. In contrast, *Myosotis brockiei* bears flowers ca 1 cm across and anthers with long filaments (ca 4 mm). When not fertile, indumentum and leaf shape are useful characters to distinguish between these two species. *Myosotis mooreana* bears oblanceolate rosette leaves and wide cauline leaves covered by a combination of arcuate, erect, antrorse and retrose hairs. *Myosotis brockiei*, in contrast, has narrowly elliptic leaves with a distinct tomentose almost greyish indumentum. Leaf shape is the most useful character to differentiate *Myosotis mooreana* from *Myosotis forsteri*. The latter species possess orbicular to broadly elliptic leaves with a well-defined petiole. The flowers of both species are very similar in size (see Table 1) but the calyx of *Myosotis forsteri* at fruiting is almost twice the size of the fruiting calyx of *Myosotis mooreana*.

##### Key to *Myosotis chaffeyorum*, *Myosotis mooreana* and other white flowered and laxly tufted species of *Myosotis* found in forest areas of New Zealand

**Table:** 

1	Rosette leaf lamina obovate to narrowly elliptic	2
–	Rosette leaf lamina orbicular to broadly elliptic	3
2	Leaf and petiole indumentum tomentose; stem erect; calyx 3.9 – 4.4 mm at fruiting; stamens fully exserted from corolla tube	*Myosotis brockiei*
–	Leaf and petiole indumentum hispid; stem ascending; calyx ca 2.8 mm at fruiting; stamens included in corolla tube	*Myosotis mooreana*
3	Petiole of rosette leaves 1.9 – 3 mm wide; stem ascending; cyme well-defined; calyx ca 5 mm long at fruiting	*Myosotis forsteri*
–	Petiole of rosette leaves 0.2 – 0.6 mm wide; stem prostrate; cyme ill-defined; calyx < 4.5 mm at fruiting	4
4	Indumentum on leaf margin, petiole and stem hispid; stem creeping, rooting at nodes	*Myosotis spathulata*
–	Indumentum on leaf margin, petiole and stem strigose; stem decumbent, no roots at the nodes	5
5	Indumentum on leaf surface hispid; pedicel 4.6 – 8.4 mm long at fruiting; calyx hairs arcuate; stamens fully exserted from corolla tube	*Myosotis matthewsii*
–	Indumentum on leaf surface strigose; pedicel 1.5 – 3 mm long at fruiting; calyx hairs straight; stamens included in corolla tube	*Myosotis chaffeyorum*

## Supplementary Material

XML Treatment for Myosotis chaffeyorum

XML Treatment for Myosotis mooreana

## Appendix 1

Representative material listed in Table 1.

*Myosotis brockiei*: New Zealand. South Island: north-west Nelson, Kahurangi National Park, base of the cliff behind Growler Rock Shelter, alt. 1003 m, 4 January 2011, C.A. Lehnebach & A.Zeller s.n. (WELT SP090249).

*Myosotis forsteri*: New Zealand, North Island: Pureora Forest Park, Whenuakura, January 2011, T.Emmitt s.n. (WELT SP092226).

*Myosotis matthewsii*: New Zealand, North Island: Warawara Forest, 8 March 2012, J.Prebble et al. JMP12-010g (WELT SP093687).

*Myosotis spathulata*: New Zealand, North Island: Waipuna Station, south end of Te Waka Range, alt. 835 m, 13 December 2011, H.Meudt et al. HMM 334 A (WELT SP090633).
